# Proficiencies of *Artemisia scoparia* against CCl_4_ induced DNA damages and renal toxicity in rat

**DOI:** 10.1186/s12906-016-1137-6

**Published:** 2016-05-27

**Authors:** Moniba Sajid, Muhammad Rashid Khan, Naseer Ali Shah, Shafi Ullah, Tahira Younis, Muhammad Majid, Bushra Ahmad, Dereje Nigussie

**Affiliations:** Department of Biochemistry, Faculty of Biological Sciences, Quaid-i-Azam University, Islamabad, Pakistan; Department of Biosciences, COMSATS Institute of Information Technology, Islamabad, Pakistan; Department of Environmental sciences, Government College Women University, Sialkot, Pakistan; Ethiopian Public Health Institute, Addis Ababa, Ethiopia

**Keywords:** *Artemisia scoparia*, DNA damages, Comet assay, Antioxidant, Lipid peroxidation, Kidneys

## Abstract

**Background:**

*Artemisia scoparia* is traditionally used in the local system of medicine in kidney disorders. This study aimed at scrutinizing the nephroprotective prospective of *A. scoparia* methanol extract against carbon tetrachloride (CCl_4_) provoked DNA damages and oxidative stress in kidneys of rat.

**Methods:**

Dried aerial parts of *A. scoparia* were powdered and extracted with methanol to obtain the viscous material (ASM). Sprague Dawley male rats (42) were grouped (7) having 6 rats in each. Group I remained untreated and Group II treated intraperitoneally (i.p) with DMSO + olive oil (1 ml/kg body weight (bw). Rats of Group III - VI were treated with CCl_4_ (1 ml/kg bw; i.p 30 % v/v in olive oil). Animals of Group IV were co-administered with 100 mg/kg bw of silymarin whereas rats of Group V and VI with 150 mg/kg bw and 300 mg/kg bw of ASM at an interval of 48 h for four weeks. Animals of Group VII were administered with ASM (300 mg/kg bw) alone. DNA damages were investigated with comet assay in renal tissues while the oxidative injuries were estimated in serum and renal tissues.

**Results:**

Co-administration of ASM to rats significantly reduced the DNA damages at 300 mg/kg dose as indicated in comet length (40.80 ± 2.60 μm), head length (34.70 ± 2.21 μm), tail length (7.43 ± 1.24 μm) and DNA content in head (88.03 ± 2.27 %) to that of CCl_4_ for comet length (63.16 ± 2.11 μm), head length (23.29 ± 1.50 μm), tail length (39.21 ± 2.81 μm) and DNA content of head (74.81 ± 2.18 %) in renal cell’s nuclei. Increased level of urea, creatinine, bilirubin, blood urea nitrogen whereas decreased concentration of proteins in serum of CCl_4_ treated animals were restored towards the normal level with co-administration of ASM. CCl_4_ injection in rats decreased the activity level of CAT, POD, SOD, GST and γ-GT and GSH contents while elevated levels of TBARS, H_2_O_2_ and nitrite contents were observed in renal tissues. A noteworthy retrieval of all these parameters and the altered histopathological observations was notified near to the normal values after treatment with both the doses of ASM.

**Conclusion:**

Results obtained suggested the therapeutic role of ASM in oxidative stress related disorder of kidneys.

## Background

World is blessed with an affluent wealth of medicinal herbs that is playing a crucial role in maintaining public health. Herbal medicines are considered significantly safer and proven a blessing for the management of a variety of ailments [1]. Increasing propensity for the efficient cure of oxidative stress related disorders has encouraged researchers towards the appraisal of medicinal plants for their antioxidant properties. Demand for medicinal plants is increasing due to the universal inclination towards advanced quality of life [2]. In the recent scenario, a great number of substantiations are being collected to depict the massive potential of medicinal plants used in various conventional systems. Bio-organic compounds have enormous therapeutic values and medicinal plants are a major source of organic constituents [3].

*Artemisia scoparia* Waldst. & Kitam., belongs to family Asteraceae (Compositae), commonly known as red stem wormwood and locally called jhahoo or jaukay. The plant flourishes well in summer season after rainfall in sandy soil of barren areas, along roads, on stony ground, waste lands and rural tracks at an altitude of 450 to 4000 m. It is an important perennial and slightly aromatic herb [4]. In subcontinent (India, Pakistan) *A. scoparia* has been used as folklore medicine for its antipyretic, anticholesterolemic, antiseptic, antibacterial, cholagogue, diuretic and vasodilator properties. *A. scoparia* is used to treat gallbladder inflammation, hepatitis and jaundice [5]. Leaves, shoots and seeds of *A. scoparia*are used in the treatment of epilepsy and sore throat by locales [6]. Mahmood et al. [7] reported that local communities use the *A. scoparia* for its astringent, carminative, aromatic, anodyne, diuretic, emmenagogue, appetizer and febrifuge properties. In addition, it is reported to be used in dyspepsia, flatulence and as vermifuge [7]. Ibrar and Hussain [8] reported that local healers utilize the aerial parts of *A. scoparia*in kidney and liver disorders. Protective effects of *A. scoparia* against acetaminophen induced hepatotoxicity have been documented [9]. Further, composition of essential oil of *A. scoparia* has been investigated and was found to be mainly composed of oxygenated monoterpenoids [10]. Singh et al. [11] studied the chemical composition and antioxidant activities of the *A. scoparia* essential oil. Habib and Waheed [12] studied the anti-nociceptive, anti-inflammatory and antipyretic effects of *A. scoparia*.

CCl_4_ is considered as perilous toxin [13]. Due to the metabolic renovation of CCl_4_ by cytochrome P-450, trichloromethyl (CCl_3_) radical and chlorine (Cl) are formed which swing the oxidant-antioxidant balance towards negative by agitating the antioxidant enzyme defense system. These free radicals afterwards, instigate the endoplasmic reticulum lipid peroxidation and start a prolonged chain reaction. Reports suggested that the effect of CCl_4_ on kidney is higher than other organs [13]. The excessive generation of free radicals causes massive damage to proteins, DNA and lipids [14]. Widespread DNA strand breaks as an effect of CCl_4_ toxicity may cause compensatory cell rejuvenation and cell death. CCl_4_ provoked oxidative stress is expected to contribute in nephrotoxicity leading to a variety of pathological conditions by inducing acute and chronic renal deteriorations [13, 14].

Different plants are a massive supply of bioactive constituents involving in the scavenging of oxidation prompting radicals [15, 16]. The natural antioxidants work as a shelter against the assaults of free radicals that can be the cause of diverse irreversible harms to the cell. The therapeutic potential of medicinal plants is attributed to their secondary metabolites. The scavenging of free radicals by the plant derived product may offer natural alternative approach to combat stress induced tissue damages [17]. *A. scoparia* is lauded with diverse therapeutic properties including renal disorders. In this study we have evaluated the protective effects of the methanol extract of *A. scoparia* aerial parts against the oxidative assault induced with CCl_4_ in kidney tissues. In this regard comet assay andthe activity level of various antioxidant enzymes of renal tissues along with biochemical analysis of serum was performed to demonstratethe protective potentialof *A. scoparia* in renal tissues.

## Methods

### Extract preparation

The plant material (aerial parts) of *A. scoparia* was collected from the main campus of Quaid-i- Azam University; Islamabad in September 2013. Specimens were authenticated by Dr. RizwanaAleem Qureshi (plant taxonomist; QAU) and a herbarium specimen (# 6335) was submitted to Herbarium, Department of Plant Sciences, Quaid-i-Azam University, Islamabad.

Shade dried aerial plant material was ground and 5 kg powder was soaked for 5 days in 10 l of methanol. Extraction was done twice and resultant filtrate was dried at 40 °C in a rotary evaporator to obtain crude methanol extract of *A.scoparia* (ASM).

### Experimental design

Male Sprague Dawley rats (150–200 g) were kept in the Primate Facility at Quaid-i-Azam University, Islamabad. The animals were placed in conventional steel cages at room temperature with standard 12 h light and dark cycle. The ethical board of Quaid-i-Azam University, Islamabad permitted the experimental protocol (Bch#264). Animals were distributed into seven groups (6 animal each group). Feed (rodent chow and tap water) was given to animals *ad libitum*. Protocol of Shyu et al. [18] was followed with few amendments to carry out this experiment. Administration of CCl_4_ (1 ml/kg of body weight in olive oil, in ratio of 30:70) was carried out intraperitoneally on alternative days for 4 weeks. Group I was control and no treatment was administered; Group II (vehicle control) was given DMSO (10 % in olive oil) orally (1 ml/kg bw). Group IIIwas administered CCl_4_(30 %) in olive oil i.p (1 ml/kg bw). Animals of Group IV were given silymarin (100 mg/kg bw) + CCl_4_. Rats of Group V and VI were administered ASM (150 mg/kg bw and 300 mg/kg bw, respectively) + CCl_4_ while Group VII was treated with ASM (300 mg/kg bw) alone.

Before dissection, all rats were kept on normal feed without any treatment for at least 24 h. Chloroform anesthesia was administered to rats and dissected from ventral side. By using 23 G1 syringes, cardiac puncture was done and blood samples were collected in falcon tubes. Falcon tubes were centrifuged at 500 × g for 15 min at 4 °C and sera were collected for biochemical analysis which included parameters such as creatinine, urea, blood urea nitrogen (BUN) and total protein estimation. Kidneys were removed, rinsed with ice cold saline to remove debris and after drying in liquid nitrogen were stored at −70 °C for tissue homogenate tests. Small parts of organs were stored in 10 % phosphate buffered formalin for comet assay and histopathological studies.

### Comet assay

Protocol of Dhawan et al. [19] was followed with slight modifications to assess the DNA damage. Sterilized slides were dipped in hot normal melting agarose (1 %) solution and allowed to solidify at room temperature. A small piece of renal tissue was placed in 1 ml of cold lysing solution and minced in to small pieces and mixed with 75 μl of low melting agarose solution. This mixture was coated on the already coated slides and a cover slip was gently placed over it. The slide was placed on icepacks for about 8–10 min. Cover slip was removed and again low melting point agarose was added and placed on ice packs for solidification. After three coating with low melting point agarose slide was again placed in the lysing solution for about 10 min and placed in refrigerator for 2 h. After electrophoresis slide was stained with 1 % ethidium bromide and visualized under fluorescent microscope. CASP 1.2.3.b image analysis software was used to evaluate the extent of DNA damage. In each sample 50–100 cells were analyzed forcomet length, head length, tail length, tail moment and DNA content in head of renal cell’s nuclei.

### Serum analysis

For the analysis of serum samples of rats, the diagnostics kits of AMP (Krenngasse 12, 8010 Graz, Australia) were used to estimate urea, creatinine, (BUN), bilirubin and total protein levels in serum samples.

### Antioxidant enzymes assessment

Kidney tissues (100 mg) of each tissue sample was homogenized in 1 ml of potassium phosphate buffer (100 mM) which contains EDTA (1 mM) and maintaining pH at 7.4. Then the centrifugation of homogenate was done at 12000 × g at 4 °C for 30 min to obtain the supernatant for following antioxidant enzyme assays.

#### Catalase (CAT) activity

For the CAT activity determination, the protocol of Chance and Maehly [20] was followed. The CAT reaction solution consisted of 625 μl of 50 mM of potassium phosphate buffer (pH 5), 100 μl of 5.9 mM H_2_O_2_ and 35 μl of the supernatant. After one minute, changes in absorbance of the reaction mixture at 240 nm were recorded. One unit of CAT activity was stated as an absorbance change of 0.01 as units/min.

#### Peroxidase (POD) activity

Activity of POD was assayed by Chance and Maehly [20] protocol with slight modifications. POD reaction solution contained 75 μlof 40 mM hydrogen peroxide, 25 μl of 20 mM guaiacol and 625 μl of 50 mM potassium phosphate buffer (pH 5.0) and 25 μl of supernatant. After an interval of one minute, change in absorbance was determined at 470 nm. One unit POD activity is equivalent to change in absorbance of 0.01 as units/min.

#### Superoxide dismutase (SOD) activity

Activity level of SOD was estimated by the protocol of Kakkar et al. [21]. By using phenazinemethosulphate and sodium pyrophosphate buffer SOD activity was assessed. Centrifugation of tissue homogenate was done at 1500 × g for 10 min and then at 10,000 × g for 15 min. Supernatant was collected and 150 μl of it was added to the aliquot containing 600 μl of 0.052 mM sodium pyrophosphate buffer (pH 7.0) and 50 μl of 186 mM of phenazinemethosulphate. In the end to initiate enzymatic reaction, 100 μl of 780 μM NADH was added. After 1 min, glacial acetic acid (500 μl) was added to stop the reaction. At 560 nm optical density was determined to enumerate the color intensity. Results were evaluated in units/mg protein.

#### Glutathione-S-transferase (GST) activity

Protocol of Habig et al. [22] was followed for the estimation of GST activity. The assay was based on formation of 1-chloro-2,4-dinitrobenzene (CDNB) conjugate. A volume of 150 μl of tissue supernatant was added to 720 μl of sodium phosphate buffer together with 100 μl of reduced glutathione (1 mM) and 12.5 μl of CDNB (1 mM). By spectrophotometer, optical density was recorded at 340 nm. Through molar coefficient of 9.61 × 10^3^/M/cm, GST activity was determined as amount of CDNB conjugate formed per minute per mg protein.

#### γ-Glutamyltranspeptidase (γ-GT)

To find out the activity of γ-GTOrlowski and Meister [23] scheme was adopted. Glutamylnitroanilide was used as substrate for verification of the activity of γ-GT. Reaction solution of γ-GT consists of an aliquot of 50 μl tissue homogenate, 250 μl of glutamylnitroanilide (4 mM), 250 μl of glycyl glycine (40 mM) and 250 μl of MgCl_2_ (11 mM) which was prepared in 185 mM TrisHCl buffer at room temperature. After 10 min of incubation, the reaction was stopped with the addition of 250 μl 25 % trichloroacetic acid. Then centrifugation was done at 2500 × g for 10 min and the optical density of collected supernatant was determined at 405 nm. Activity of γ-GT was determined as nMnitroaniline formed per min per mg protein by the use of molar extinction coefficient of 1.75 × 10^3^/M/cm.

### Estimation of biochemical parameters

#### Reduced glutathione (GSH) estimation

Quantity of GSH in kidney tissues was assessed following the protocol of Jollow et al. [24]. Precipitation of tissue homogenate (500 μl) was carried out by the addition of (500 μl) 4 % sulfosalicylic acid. After 1 h of incubation at 4 °C the reaction mixture was centrifuged for 20 min at 1200 × g. An aliquot of 33 μlof the supernatant was added to 900 μl of 0.1 M potassium phosphate buffer (pH 7.4) and 66 μl of 100 mM of 5,5’-dithio-bis(2-nitrobenzoic acid (DTNB). Reaction of GSH with DTNB produced a yellow colored derivative 5’-thio-2-nitrobenzoic acid (TNB). The optical density of the reaction mixture was recorded at 412 nm. The GSH activity was expressed as μM GSH/g tissue.

#### Lipid peroxidation assay (TBARS)

Protocol of Iqbal et al. [25] was adopted with slight modifications for the assessment of lipid peroxidation. The reaction mixture consisted of 290 μl of 0.1 M phosphate buffer (pH 7.4), 10 μl of 100 mM ferric chloride, 100 μl of 100 mM ascorbic acid, and 100 μl of homogenized sample. After 1 h incubation of the mixture at 37 °C in shaking water bath500 μl of trichloroacetic acid (10 %) was added to inhibit the reaction. Then 500 μl of 0.67 % thiobarbituric acid was added andthe reaction tubes were placed in water bath for 20 min. After that the tubes were placed in crushed ice bath for 5 min and centrifugation was done at 2500 × g for 12–15 min. Absorbance of the supernatant was recorded at 535 nm. By using molar extinction coefficient of 1.56 × 10^5^/M/cm, results were calculated as nM of TBARS formed per min per mg tissue at 37 °C.

#### Protein assessment

Procedure of Lowry et al. [26] was followed in order to find the total soluble proteins within the tissues. For this purpose, 100 mg of organ was weighed and homogenization was done in potassium phosphate buffer. Homogenized mixture was centrifuged at 4 °C at 10,000 × g for 15–20 min to obtain the supernatant. Alkaline solution 1 ml was added in 0.1 ml of supernatant and mixed vigilantly with the help of vortex machine. Then the incubation was done for 30 min. Afterwards the change in absorbance was calculated at 595 nm. Bovine serum albumin (BSA) curve was used to find out the concentration of serum proteins in the sample.

#### Hydrogen peroxide (H_2_O_2_)assay

Estimation of hydrogen peroxide was done by following Pick and Keisari [27] protocol. The H_2_O_2_ horseradish peroxidase enzyme brought about the oxidation of phenol red. In the reaction mixture, 500 μl of 0.05 M phosphate buffer (pH 7),100 μl of homogenate was added along with 100 μl of 0.28 nM phenol red solution, 250 μl of 5.5 nM dextrose and horse radish peroxidase (8.5 units) was added. Incubation was done at room temperature for 60 min. A volume of 100 μl of NaOH (10 N) was added to stop the reaction. Then mixture tubes were centrifuged for 5–10 min at 800 × g. By spectrophotometer the absorbance of the collected supernatant was measured against reagent as a blank at 610 nm. Production of H_2_O_2_ was measured as nM H_2_O_2_/min/mg tissue on the basis of standard curve of H_2_O_2_ oxidized phenol red.

#### Nitrite assay

For the execution of nitrite assay, Griess reagent was utilized [28]. The homogenate was treated with equal volume of 100 μl of both 5 % ZnSO_4_ and 0.3 M NaOH. Centrifugation was done at 6400 × g for 15–20 min. Afterwards 20 μl supernatant was mixed with 1.0 ml of Griess reagent in cuvette and at 540 nm change in color was determined. Griess reagent 1 ml was used as a blank in the spectrophotometer. Standard curve of sodium nitrite was utilized for evaluation of nitrite concentration in renal tissues.

### Histopathological examination

For histopathological examination, a fixative containing absolute alcohol (85 ml), glacial acetic acid (5 ml) and 40 % formaldehyde (10 ml) was used to fix renal tissues. For slides preparation, thin sections of fresh tissues of kidney about 3–4 μm were used. The hematoxylin-eosin stain was used for staining purpose and for histopathological study a light microscope (DIALUX 20 EB) at magnification of 40X was used.

### Statistical analysis

The values were expressed as mean ± standard deviation. For *in vivo* studies, the consequences of different treatments given to animals were evaluated by Kruskal-Wallis test based on non-parametric analysis of variance by using the computer software Statistix 8.1. Multiple comparisons among various treatments were made at P-value ≤0.05.

## Results

The present study was designed to inspect the protective potential of crude methanol extract of *A.scoparia* against CCl_4_ induced renal toxicity at biochemical, histological and molecular level in rats. For this purpose different parameters were analyzed including serum profile, antioxidant enzymatic levels, morphological changes provoked by CCl_4_ in histopathologyand genotoxicity analysis was determined by comet assay.

### Comet assay

Damage in DNA and defensive effect of *A.scoparia* on CCl_4_ intoxicated kidney cells of rats was assessed by comet assay. Consequence of ASM on DNA damages in kidney cells of Sprague Dawley rats is mentioned in Table 1. In control group a small number of comets with very tiny tail length and larger number of cells with intact DNA were observed. In CCl_4_ treated group DNA damage was induced and resulted in significant (*P* < 0.05) increase in the comet length and tail length while decrease (*P* < 0.05) in head length was determined in kidney cells (Table 1). Co-administration of silymarin and ASM ameliorated the toxicity to DNA and the comet values were restored towards the control level. ASM reduced the DNA damagesat the lower as well as higher dose. Highest dose of ASM produced more conspicuous protective effects and restored the comet values towards the control group. Treatment of ASM alone to rats resulted in non-significant increase in tail length and consequently the comet length in kidney cells as compared to control.

**Table 1:** Protective effects of ASM on comet parameters in renal cells

Group	Comet length (μm)	Head length (μm)	Tail length (μm)	% DNA in head	% DNA in tail	Tail moment (μm)
Control	41.48 ± 2.63	35.04 ± 1.81	6.61 ± 1.03	91.33 ± 2.21	9.53 ± 1.13	0.51 ± 0.031
Olive oil + DMSO	40.85 ± 2.39	34.58 ± 1.78	6.36 ± 0.68	90.36 ± 2.43	10.11 ± 1.04	0.61 ± 0.023
CCl_4_	63.16 ± 2.11aab	23.29 ± 1.50ab	39.21 ± 2.81ab	74.81 ± 2.18ab	25.28 ± 1.40ab	0.64 ± 0.019
CCl_4_ + Sily (100)	41.54 ± 2.56c	34.94 ± 2.06c	6.35 ± 0.71c	90.46 ± 2.52c	9.70 ± 1.19bc	0.44 ± 0.021c
CCl_4_ + ASM (150)	46.76 ± 1.74	28.22 ± 2.21	18.78 ± 1.62	84.53 ± 2.32	15.95 ± 1.42	0.60 ± 0.025
CCl_4_ + ASM (300)	40.80 ± 2.60c	34.70 ± 2.21c	7.43 ± 1.24c	88.03 ± 2.27c	11.66 ± 1.45c	0.45 ± 0.018c
ASM (300)	43.20 ± 3.21	34.67 ± 2.23c	9.26 ± 1.57c	89.98 ± 1.95c	10.35 ± 1.16c	0.11 ± 0.024bc

Values are expressed as mean ± SD (*n* = 6), Sily. Silymarin; ASM: *A. scoparia* methanol extract. Means with letter “a” indicate significant difference from control, “b” from vehicle control and “c” from CCl_4_ treated group according to Kruskal-Wallis test at *P* < 0.05

**Fig. 1:**
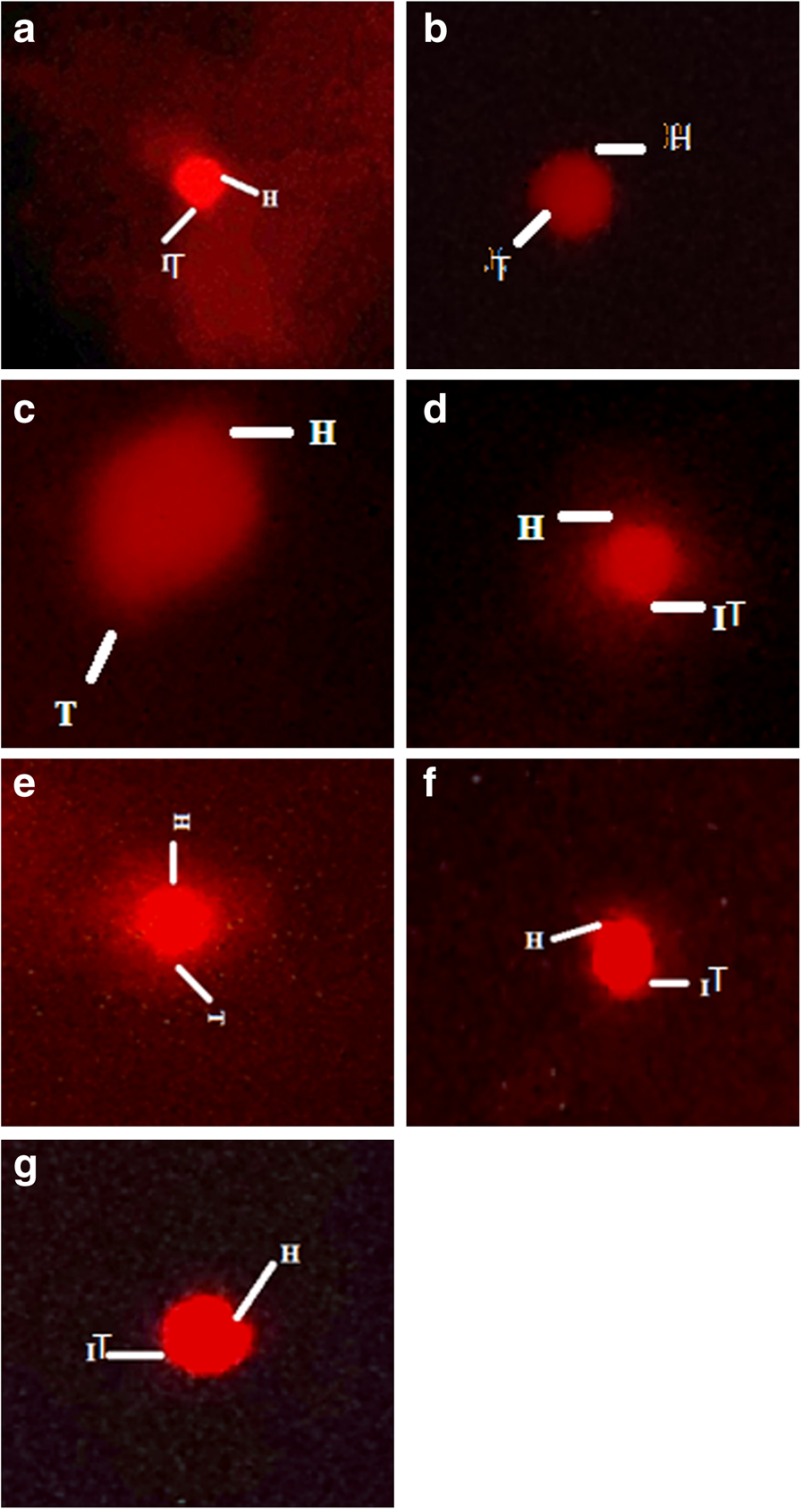
Fluorescence photomicrograph of kidney cells and protective outcome of ASM on genotoxicity. **a** Control group; **b** Vehicle control, **c** CCl_4_(1 ml/kg b.w., i.p., 30 % in olive oil group; **d** CCl_4_+ silymarin (100 mg/kg); **e** CCl_4_ + ASM (150 mg/kg); **f** CCl_4_ + ASM (300 mg/kg); **g** ASM (300 mg/kg). ASM; *A. scoparia* methanol extract; H, Comet head; T, Comet tail

In CCl_4_ intoxicated group a sharp decrease in concentration of DNA in comet head whereas an increase in comet tail was exhibited in kidney cells relative to control (Table 1). A notable increase (*P* < 0.05) in tail moment was also determined in kidney cells of CCl_4_ treated group as against the control group. Treatment of ASM along with CCl_4_ remarkably restored the above parameters in renal cells of rat. Concentration of DNA in head was sharply enhanced in head of comet along with significant (*P* < 0.05) decrease in tail moment in kidney cells of rat treated with the highest dose of ASM and CCl_4_. Treatment of ASM alone to rats did not influence the concentration of DNA in head and tail of comet in renal cells but significantly (*P* < 0.05) reduced the tail moment as compared to the control group. Figure 1 depicts microphotograph of control and CCl_4_ intoxicated kidney cells and protective potential of *A. scoparia* on genotoxicity.

### Protective outcome of ASM on serum profile of rats

Protective approach of ASM on serum markers against CCl_4_ induced renal toxicity in rats is shown in Table 2. The concentration of urea, bilirubin, creatinine and BUN were significantly enhanced (*P* < 0.05) in CCl_4_ treated rats while level of total proteins was decreased in serum. The toxic effects of CCl_4_ were diminished with ASM co-administration and the altered level of above parameters was restored towards the control level.

**Table 2:** Protective outcomes of ASM on serum markers

Treatment	Urea (mg/dl)	Creatinine (mg/dl)	Bilirubin (mg/dl)	Serum proteins (mg/dl)	BUN (mg/dl)
Control	29.97 ± 2.43	0.49 ± 0.025	0.39 ± 0.024	6.15 ± 0.19	13.15 ± 1.49
Olive oil + DMSO	28.00 ± 2.10	0.43 ± 0.018	0.42 ± 0.027a	6.07 ± 0.09	12.67 ± 1.61
CCl_4_	72.02 ± 1.96	1.52 ± 0.064b	1.97 ± 0.077ab	3.82 ± 0.12ab	30.49 ± 2.28ab
CCl_4_ + Sily (100)	30.03 ± 1.83	0.41 ± 0.034 ac	0.88 ± 0.030a	5.74 ± 0.21	13.16 ± 1.18c
CCl_4_ + ASM (150)	23.37 ± 2.05c	0.61 ± 0.035b	0.92 ± 0.022a	5.05 ± 0.18ab	23.78 ± 1.76b
CCl_4_ + ASM (300)	26.32 ± 2.06c	0.56 ± 0.024	0.83 ± 0.023	5.66 ± 0.24	19.95 ± 1.97
ASM (300)	28.79 ± 1.54	0.43 ± 0.035c	0.41 ± 0.023c	5.95 ± 0.23c	12.89 ± 1.26c

Mean ± SD (*n* = 6), Sily: Silymirin; ASM: *A. scoparia* methanol extract. Means with letter “a” indicate significant difference from control, “b” from vehicle control and “c” from CCl_4_ treated group according to Kruskal-Wallis test at *P* < 0.05

### Protective effects of ASM on renal antioxidant enzymes

The levels of CAT, POD and SOD in kidney homogenates are given in Table 3. Due to CCl_4_ intoxication in rat the level of CAT, POD and SOD was significantly (*P* < 0.05) decreased in renal tissues as compared to control animals. A noteworthy decrease (*P* < 0.05) in the values of GST and γ-GT was monitored in CCl_4_ intoxicated rats. The deleterious effects of CCl_4_were diminished in renal tissues of rat simultaneously treated with ASM. The protective effects of ASM towards the antioxidant enzymes were exhibited at both doses. ASM at its maximum dose to ratsquite comprehensively restored the level of above enzymes. Treatment of rats with ASM alone did not alter the activities of CAT, SOD, POD, GST and γ-GT in renal tissues to that of the control animals.

**Table 3:** Protective effects of ASM on renal antioxidant enzymes

Treatment (mg/kg bw)	CAT (U/min)	POD (U/min)	SOD (U/mg Protein)	GST (nM/min/mg protein)	γ-GT (nM/min/mg protein)
Control	5.48 ± 0.16	7.42 ± 0.57	3.24 ± 0.27	21.55 ± 1.48	81.47 ± 2.97
Olive oil + DMSO	4.57 ± 0.50	6.85 ± 0.36	2.76 ± 0.25	22.48 ± 1.68	83.10 ± 2.53
CCl_4_	1.20 ± 0.13a	0.93 ± 0.12ab	0.84 ± 0.13ab	08.95 ± 0.91ab	40.88 ± 2.78ab
CCl_4_ + Sily (100)	4.18 ± 0.23	6.71 ± 0.42	2.51 ± 0.17	21.11 ± 1.42c	80.11 ± 2.68c
CCl_4_ + ASM (150)	2.43 ± 0.20a	4.74 ± 0.30	1.89 ± 0.17a	16.58 ± 1.14b	61.11 ± 2.78b
CCl_4_ + ASM (300)	4.41 ± 0.31	6.81 ± 0.48	2.60 ± 0.21	20.50 ± 1.91	69.06 ± 3.96
ASM (300)	5.16 ± 0.32c	6.93 ± 0.46c	3.15 ± 0.27c	20.98 ± 1.90c	83.00 ± 2.84c

Mean ± SD (*n* = 6), Sily: Silymirin; ASM: *A. scoparia* methanol extract. Means with letter “a” indicate significant difference from control, “b” from vehicle control and “c” from CCl_4_ treated group according to Kruskal-Wallis test at *P* < 0.05

### Protective effects of ASM on renal biochemicals

Table 4 illustrates the protective effect of ASM on renal proteins, H_2_O_2_ and nitrite content. Due to CCl_4_ intoxication the level of proteins in renal tissues of rat was drastically decreased (*P* < 0.05) while the level of H_2_O_2_ and nitrite content was enhanced (*P* < 0.05) to that of the control rats. This anomaly was removed with simultaneous application of ASM by patching up the cellular damage. The extent of renal damage with CCl_4_ intoxication was determined by estimating the concentration of GSH and TBARS in renal tissues of rat. In CCl_4_ treated rats an increase (*P* < 0.05) in TBARS was observed and this escalation was significantly (*P* < 0.05) removed by co-treatment with both doses of ASM. Drastic decrease in GSH in renal tissues was determined with CCl_4_ treatment to rats. Amelioration in the toxic effects on GSH was determined by the co-administration of ASM. However, more GSH content was displayed at the higher dose of ASM. Administration of ASM alone at 300 mg/kg did not affect (*P* > 0.05) the concentration of H_2_O_2_, nitrite, TBARS and GSH as compared to the control group.

**Table 4:** Protective outcome of ASME on biochemical parameters in rat kidney

Treatment	Proteins (μg/mg tissue)	GSH (μM/g tissue)	TBARS (nM/min/mgprotein)	H_2_O_2_ (μM/ml)	Nitrite (μM/ml)
Control	2.41 ± 0.16	19.30 ± 1.35	22.35 ± 2.22	0.26 ± 0.02	51.97 ± 3.11
Olive oil + DMSO	2.55 ± 0.09	18.51 ± 2.03	22.08 ± 1.85	0.24 ± 0.02	50.41 ± 3.04
CCl_4_	1.06 ± 0.10ab	3.75 ± 0.25ab	44.52 ± 3.58ab	0.62 ± 0.02ab	91.74 ± 3.84ab
CCl_4_ + Sily (100)	2.43 ± 0.14c	18.36 ± 1.64c	23.55 ± 2.16	0.26 ± 0.02bc	52.61 ± 3.16
CCl_4_ + ASM (150)	1.68 ± 0.18b	09.45 ± 0.94a	25.45 ± 1.43	0.42 ± 0.02bc	54.12 ± 3.05
CCl_4_ + ASM (300)	2.14 ± 0.12	14.25 ± 1.25	22.96 ± 1.80c	0.31 ± 0.04	52.85 ± 3.03
ASM (300)	2.30 ± 0.17	17.83 ± 1.19c	22.33 ± 1.83c	0.30 ± 0.02	51.49 ± 2.74c

Mean ± SD (*n* = 6), Sily: Silymirin; ASM: *A. scoparia* methanol extract. Means with letter “a” indicate significant difference from control, “b” from vehicle control and “c” from CCl_4_ treated group according to Kruskal-Wallis test at *P* < 0.05

### Protective effects of ASM on histology of renal tissues

**Fig. 2:**
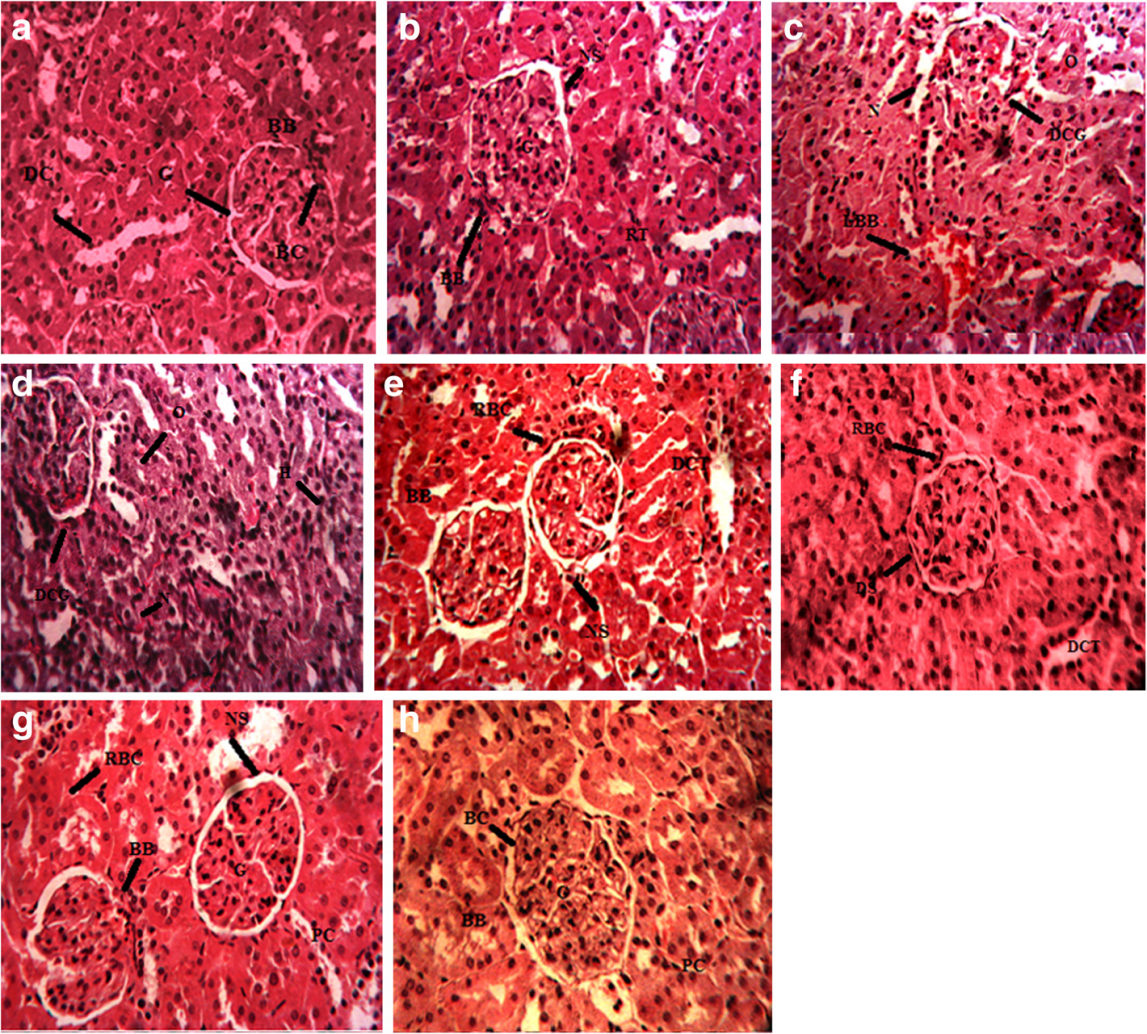
Protective outcome of ASM on histology of renal tissues (40X magnification). **a** Control group; **b** Vehicle control, **c**, **d** CCl_4_ (1 ml/kg b.w., i.p., 30 % in olive oil group; **e** CCl_4_ + silymarin (100 mg/kg); **f** CCl_4_ + ASM (150 mg/kg); **g** CCl_4_ + ASM (300 mg/kg); **h** ASM (300 mg/kg). G, glomerulus; BC, Bowman’s capsule; O, interstitial edema; H, glomerular hypocellularity; N, necrosis of epithelium; PC, proximal convoluted tubule; BB, brush border; DC, distal convoluted tubule; DCT, dilated proximal convoluted tubule; RT, renal tubule; LBB loss of brush border; DCG, degenerative changes in glomerulus; RBC, regenerating Bowman’s capsule; NS, normal space between Bowman’s capsule and glomerulus; DS, decreased space between Bowman’s capsule and glomerulus

Histological assessment of renal tissues was done after hematoxylin-eosin staining underneath the light microscope. Renal tissues from each experimental group were studied as shown in Fig. 2. Typical regular morphology of kidney tissues was examined in rats of control and vehicle groups (A and B). Significant histological changes were observed in both cortex and medulla in kidney tissues of CCl_4_ treated rats (C and D). CCl_4_ prompted the induction of reactive oxygen species which in turn provoked a high degree of harm to the cortical region of kidneys. Cortex was more rigorously affected due to the CCl_4_ intoxication as compared to medulla. In CCl_4_ treated rats the renal segments demonstrated tubular dilation, interstitial fibrosis, tubular deterioration, glomerular atrophy, glomerular hypertrophy, obliteration of Bowman’s capsule of nephrons and clogging in capillaries. Treatment with low dose (150 mg/kg bw) had narrowed the chronic damages and the high dose of ASM (300 mg/kg bw) markedly preserved the normal morphology of kidneys and depicted normal glomerular, tubular structure and averted the interstitial edema and capillary clogging. However, histology of silymarin treated group was closely related with the normal group.

## Discussion

Free radicals are considered to be involved in DNA damages, lipid peroxidation and protein injuries leading to acute or chronic renal disorders. Toxic manifestations of the reactive species can be ameliorated by taking the diet rich in antioxidant metabolites. Aerial parts of *A. scoparia* are composed of diverse metabolites having antioxidant abilities [9, 11]. The present investigation was carried out to demonstrate the nephroprotective effects of *A.scoparia* extract against CCl_4_ mediated renal oxidative trauma. The defensive outcome of *A.scoparia* was evaluated by estimating the serum markers level and by measuring activity levels of antioxidant enzymes in renal tissues. Further, the levels of GSH, TBARS, nitrite and H_2_O_2_were determined in renal tissues along with DNA damages and histopathological alterations.

To appraise the DNA damages induced with reactive species in renal tissues comet assay was performed. Comet assay is a responsive and adaptable technique which deciphers the DNA strand breakage at the single cell level [29]. In current study significant increase in tail moment, tail length, head length, comet length, % DNA in tail was recorded with CCl_4_ administration in renal cells of rat. In our results, long tail length of comet reveals high extent of DNA damage in CCl_4_ treated renal cells of rats. Comet tail length is an investigative of DNA fragmentation in any cell variety studied by comet assay. The altered comet parameters were reversed towards the control level by the co-administration of ASM and the DNA protective effect was more pronounced at the higher dose of ASM. These results suggest that *A. scoparia* is a worthy candidate to inhibit the DNA damage in renal tissues.

Our study displayed that CCl_4_ intoxication made a remarkable increase in urea, creatinine, bilirubin and BUN levels while serum protein was considerably decreased. Enhanced creatinine and urea level in serum reflects the impaired renal function and/or injured nephrons. Oxidative stress induced with CCl_4_ is not restricted to a single organ but it provides a link with other organs as well. Decrease in serum protein might occur as an effect of CCl_4_ on the synthesis of proteins from hepatocytes along with enhanced proteinuria. This study coincides with the findings of Irshaid et al. [30] where they also characterized that the level of urea and creatinine significantly increased in serum due to alloxan induced toxicity. However these aberrations in normal levels of renal parameters were diminished by treatment with *A. sieberi* extract.

To assess the activity level of antioxidant enzymes is also very important to monitor the injuries induced with CCl_4_in renal tissues. In the present study toxicity of CCl_4_ provoked a noteworthy decrease in the activity level of CAT, POD, SOD, GST and γ-GT in renal tissues of rat. However, the concentration of H_2_O_2_was increased in renal tissues of rat treated with CCl_4_. The enhanced contents of H_2_O_2_ in renal tissues were indicative of the compromised activity of antioxidant enzymes [15, 17]. SOD catalyzes the conversion of superoxide ions in to H_2_O_2_ which then subsequently decomposed by CATinto oxygen and water. The co-administration of ASM to CCl_4_ treated rats exhibited repairing potential towards the injuries induced with CCl_4_. The protective effects of ASM led to an increase in the activity level of antioxidant enzymes and with concomitant decrease in H_2_O_2_ content of renal tissues. This study suggests the presence of protective phytoconstituents in the plant extract which diminished the oxidative assault induced with CCl_4_ in renal tissues of rat.

The present study also indicated the toxic effects of CCl_4_ by enhancing the concentration of lipid peroxidation assay (TBARS) and nitrite content while decreasing the GSH in renal tissues of rat. Glutathione peroxidase (GSH-Px) effectively scavenges the H_2_O_2_ and other organic hydroperoxides with the support of GSH. Diminution of GSH level in a tissue can occur due to utilization of NADPH or GSH in removal of peroxides. Detoxification of peroxides usually occurs at the expense of GSH which is oxidized into GSSG (oxidized glutathione) [31]. Lipid peroxidation assay (TBARS) are generated due to peroxidation of polyunsaturated fatty acids which arethe final metabolites of this chain of reactions and measured as biomarkers of oxidative stress [32]. Acidic conditions prevailed at the CCl_4_-induced injured areas augment the synthesis of nitrite from nitric oxide which subsequently changes into peroxynitrite by interaction with superoxide radicals. The compromised activity of the antioxidant system might provoke the generation of secondary reactive species having a more active role in the lipid peroxidation. Administration of ASM in CCl_4_ intoxicated rats alleviated the toxic effects of CCl_4_ and restored the concentration of GSH, TBARS and nitrite content towards the level of control rats. The results obtained in this study are supported by earlier investigations [33] where the extract of *A. judaica* restored the content of these parameters in hyperlipidemia and hyperglycemic rats.

It is conceivable from the present results that CCl_4_ treatment arbitrates lipid peroxidation of lipid structure of renal tissues which stimulates and sustains subcellular injuries as depicted in histopathological inspection. The current study has revealed that kidneys of CCl_4_ treated rats have specified morphological findings such as disruption of kidney glomeruli, interstitial fibrosis, proximal and distal tubules’ edema, loss of brush border, glomerular atrophy and necrosis of epithelium. These severe adaptations were not spotted in animals co-treated with ASM, suggesting the protective outcomes of *A.scoparia* in deteriorating CCl_4_ activated morphological progressions. However, our results do not comply with the studies of Noori et al. [34] where ethanolic extract of *A. deserti* flowering tips (200 mg/kg bw) induces alteration in the histopathology and enhanced the creatinine level in serum of Wistar rats. These differences might arise due to difference in plant species and possibly the composition of secondary metabolites stored.

## Conclusion

Our study suggests that *A. scoparia*has the ability to ameliorate the CCl_4_ provoked renal injuries and has restored the serum markers, DNA damages, level of enzymatic activityand histopathological alterations. The protective effects of ASM might possibly be associated with its antioxidant properties.

## Abbreviations

ALT, alanine transaminase; ASM, *Artemisia scoparia* methanol extract of aerial parts; AST, aspartate transaminase; BUN, blood urea nitrogen; CAT, catalase; CCl_4_, carbon tetrachloride; GSH, rerduced glutathione; GSR, glutathione reductase; GST, glutathione-S-transferase; POD, peroxidase; SOD, superoxide dismutase; TBARS, thiobarbituric acid reactive substances
